# Enhanced torsional actuation and stress coupling in Mn-modified 0.93(Na_0.5_Bi_0.5_TiO_3_)-0.07BaTiO_3_ lead-free piezoceramic system

**DOI:** 10.1080/14686996.2016.1254569

**Published:** 2017-01-09

**Authors:** Pelin Berik, Deepam Maurya, Prashant Kumar, Min Gyu Kang, Shashank Priya

**Affiliations:** ^a^ Bio-inspired Materials and Devices Laboratory (BMDL), Center for Energy Harvesting Materials and Systems (CEHMS), Virginia Tech, Blacksburg, VA, USA

**Keywords:** Lead-free, piezoceramic, shear-mode, torsion, actuation, 10 Engineering and structural materials; 107 Glass and ceramic materials; 202 Dielectrics/Piezoelectrics/Insulators, 304 Powder processing/Sintering, 400 Modeling/Simulations

## Abstract

This paper is concerned with the development of a piezoelectric *d*_15_ shear-induced torsion actuator made of a lead-free piezoceramic material exhibiting giant piezoelectric shear stress coefficient (*e*_15_) and piezoelectric transverse shear actuation force comparable to that of lead-based shear-mode piezoceramics. The Mn-modified 0.93(Na_0.5_Bi_0.5_TiO_3_)-0.07BaTiO_3_ (NBT-BT-Mn) composition exhibited excellent properties as a torsional transducer with piezoelectric shear stress coefficient on the order of 11.6 C m^–2^. The torsional transducer, consisting of two oppositely polarized NBT-BT-Mn *d*_15_ mode piezoceramic shear patches, provided a rate of twist of 0.08 mm m^–1^ V^–1^ under quasi-static 150 V drive. The high value of piezoelectric shear *d*_15_ coefficient in NBT-BT-Mn sample further demonstrated its potential in practical applications. These results confirm that the lead-free piezoceramics can be as effective as their lead-based counterparts.

## Graphical abstract

**Figure:**
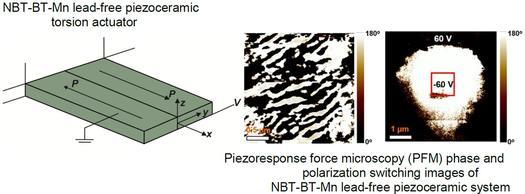

## 1. Introduction

The continued research emphasis on piezoelectric materials is related to their extensive range of applications including energy harvesting, structural health monitoring, vibration damping, underwater acoustics and actuation. Lead-based piezoelectric materials exhibit excellent actuation and sensing capabilities. However, with rising concern about the presence of lead in consumer applications there is a continuous emphasis towards finding a lead-free alternative that can meet the application requirements. In the last decade, several lead-free piezoelectric materials have been investigated to address this need,[1–4] and out of these investigations, Na_0.5_Bi_0.5_TiO_3_–BaTiO_3_ (NBT-BT) based compositions have emerged as a potential candidate due to their high *E*-field induced strain, piezoelectric response and ferroelectric polarization.[5–8]

In the past, significant research has been conducted to analyze the extension/bending effect of the piezoelectric actuators; however, a limited number of investigations have been performed on developing the piezoelectric shear actuators. Piezoelectric shear actuation is induced by applying an electric field perpendicular to the axis of polarization. The advantage of using shear mode can be seen from the fact that piezoelectric *d*_15_ shear coefficient is higher than the transversal and longitudinal piezoelectric coefficients (*d*_31_ and *d*_33_) respectively. Similarly, the value of shear electromechanical coupling coefficient (*k*_15_) is higher than that of *k*_31_ and *k*_33_. For example, commercial EC-65 material from Exelis Inc. (McLean, VA, USA) exhibits *d*_33_ = 380 pC/N, *d*_31_ = -−173 pC/N, and *d*_15_ = 584 pC/N. Berik and Benjeddou [9] have reported experimental and numerical results for the use of lead-based piezoceramic *d*_15_ shear actuators as the core of a sandwich structure. In their work, experiments on composite piezoelectric *d*_15_ shear transducers were presented for three benchmarks that differed in length and polarization configurations, demonstrating the nonlinearity for shear actuation mechanism (SAM). Butz et al. [10] conducted a numerical analysis on a bimorph torsional transducer composed of two adjacent piezoceramic rods (PZT-5H) bonded to each other along their width with polarization axes opposite to each other in the same plane. In that study, a bimorph *d*_15_ torsion transducer was theoretically introduced [10] which was later experimentally demonstrated by Berik and Benjeddou [11] using a sandwich patch actuator made of axially oppositely poled (OP) two adjacent rows of triple PZT PIC 255 patches and glass fiber reinforced polymer (GFRP) face covering layers. The torsion actuation mechanism (TAM) [11,12] has been further analyzed [13] by conducting quasi-static and static experiments for a benchmark that was validated through Saint Venant-type solutions presented by Krommer et al. [14,15]. Experimental visualization of the *d*_15_ shear-induced torsion deformation through the quasi-static scanning laser deflection measurements has been investigated, setting the reference condition for further development.[13]

Following on from the first author’s previous works on lead-based piezoceramic *d*_15_ shear-induced torsion actuators [11,13] and the study on the effects of manganese additive on piezoelectric properties of BNT-BT ferroelectric ceramics,[16] the present study contributes:

• the first lead-free piezoceramic *d*_15_ shear-induced torsion actuator; previously lead-based piezoceramic torsion actuators were analyzed;
• analysis of piezoelectric *d*_15_ shear-mode properties of NBT-BT-Mn lead-free piezoceramics exhibiting improved piezoelectric shear response, transverse shear actuation force and fulfilling the needs of a high performance piezoceramic torsion actuator;
• piezoresponse force microscopy (PFM) investigation of the domain morphology and the discussion of the effect of domain size on the piezoelectric response of NBT-BT-Mn piezoceramics;
• systematic evaluation of a lead-free piezoceramic NBT-BT-Mn torsion actuator in terms of generated angle of twist using laser scanning vibrometery, finite element (FE) simulations and 3D Saint-Venant type exact mathematical solutions;
• experimental validation of Saint-Venant type torsion solutions;[14,15] previously their verifications were conducted only through FE analysis;
• discussion of piezoelectric nonlinearity of *d*_15_ shear-mode NBT-BT-Mn piezoceramics; and
• an update to the process of FE simulations and mathematical solutions by taking into account the piezoelectric nonlinearity of *d*_15_ shear-mode piezoceramics; in earlier works,[11,13] the verifications were conducted only through linear FE analysis.

Hereafter, we demonstrate a lead-free NBT-BT-Mn piezoelectric ceramic material with enhanced piezoelectric shear stress *e*_15_ coupling coefficient and utilize it to fabricate a high performance lead-free piezoceramic *d*_15_ shear-based torsion actuator. Systematic evaluation of the piezoceramic torsion transducer based on piezoelectric *d*_15_ shear mode was conducted using a laser scanning vibrometer and results are compared with the Saint-Venant type exact 3D solutions [15] and three-dimensional FE simulations. The rate of the twist produced by torsion transducer was evaluated under quasi-static applied voltage. These results demonstrate that the lead-free torsion transducer reported here can be a viable alternative for lead-based ones.

## 2. Experimental procedure

**Figure 1.**
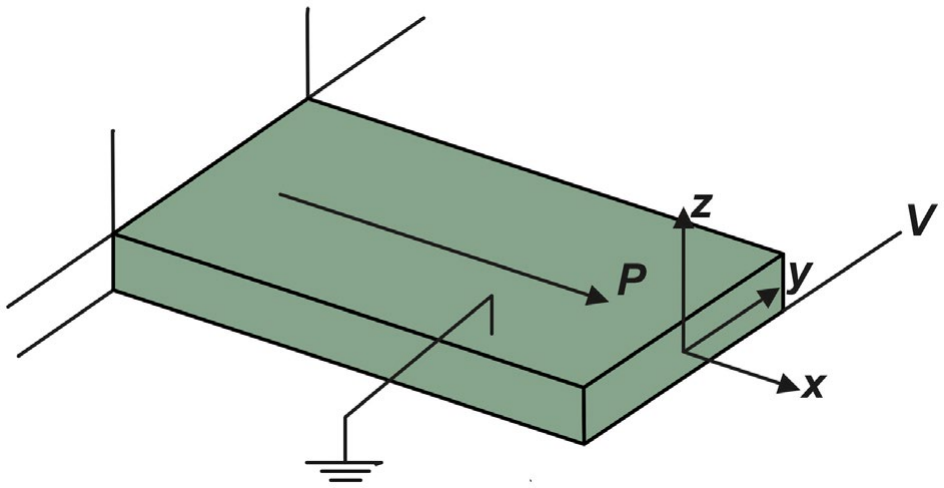
*d*_15_ shear polarization direction of the lead-free piezoceramic patches.

A morphotropic phase boundary (MPB) 0.93(Na_0.5_Bi_0.5_TiO_3_)-0.07BaTiO_3_ (NBT-BT) composite was synthesized using the conventional solid state reaction method. Stoichiometric amounts of high purity powders of Na_2_CO_3_, TiO_2_, BaCO_3_ and Bi_2_O_3_ (99.5%, Alfa Aesar, Ward Hill, MA, USA) were mixed and ball milled for 24 h with yttria-stabilized ZrO_2_ balls in ethanol media in a polyethylene bottle. The ball milled powders were further subjected to two-step calcination at 800–900 °C for 2 h. The calcined powder was ball milled for 48 h followed by palletization and sintering at 1200 °C for 2 h. In order to synthesize Mn-doped NBT-BT piezoceramic material, 0.08 wt% MnO_2_ (99.9%, Alfa Aesar) was added into the calcined NBT-BT powder.[16] NBT-BT-0.08 wt% Mn is referred as NBT-BT-Mn in the manuscript. Figure 1 shows the *d*_15_ shear polarization direction of the lead-free NBT-BT-Mn piezoceramic rectangular patch.

**Figure 2.**
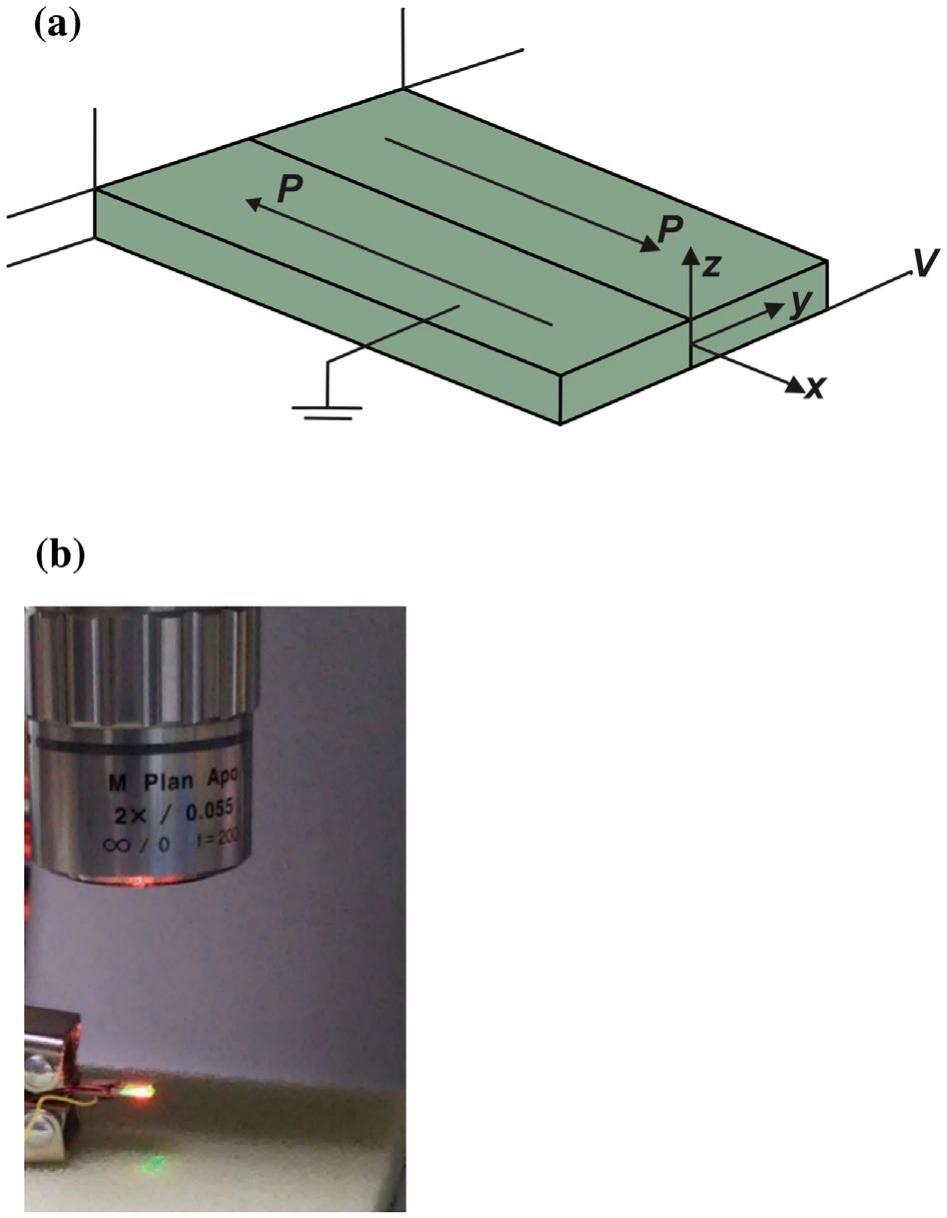
(a) Schematic of the benchmark sample. (b) Experimental setup for characterization of the benchmark using laser vibrometer.

The torsion transducer was fabricated using two identical lead-free NBT-BT-Mn *d*_15_ shear-mode piezoceramic patches with opposite (OP) polarization direction (Figure 2). The dimension of the reference sample was fixed to be 11 × 7.20 × 0.54 mm^3^. The rectangular patches were bonded to each other using copper-based adhesive material. The torsion actuation experiments were conducted using a laser scanning vibrometer (Polytec MSA-500, Waldbronn, Germany) by applying varying sinusoidal excitation voltages ranging from 30 V to 180 V at 10 Hz across the top and bottom electrodes of the piezoceramic patches in order to determine the rate of twist. The samples were mounted in the cantilever configuration and subjected to an electric voltage (V) such that the applied electric field was perpendicular to the polarization direction.

Since the polarization direction is along the x-axis, the induced actuation strains (Eigenstrains) by the right piezoceramic shear patch in Figure 2(a) can be expressed as:

(1) $${\left\{\begin{array}{c} s_{xx}\\ s_{yy}\\ s_{zz}\\ \gamma_{yz}\\ \gamma_{xz}\\ \gamma_{xy} \end{array}\right\}}^{*}=\left[\begin{array}{ccc} d_{33} & 0 & 0\\ d_{31} & 0 & 0\\ d_{31} & 0 & 0\\ 0 & 0 & 0\\ 0 & 0 & d_{15}\\ 0 & d_{15} & 0 \end{array}\right]\left\{\begin{array}{c} E_{x}\\ E_{y}\\ E_{z} \end{array}\right\}=\left\{\begin{array}{c} d_{33}E_{x}\\ d_{31}E_{x}\\ d_{31}E_{x}\\ 0\\ d_{15}E_{z}\\ d_{15}E_{y} \end{array}\right\}$$

where *s*_*xx*_ is the strain along the *x* direction, *s*_*yy*_ is the strain along the *y* direction, *s*_*zz*_ is the strain along the *z* direction, $\gamma_{yz}$ is the shear strain in the *yz* plane, $\gamma_{xz}$ is the shear strain in the *xz* plane and $\gamma_{xy}$ is the shear strain in the *xy* plane.

The transverse shear actuation strain $\gamma_{\text{xz}}^{*,\text{r}}=d_{15}E_{z}$ produced by the right piezoceramic patch is the dominant strain. Due to the opposite polarization direction, the left piezoceramic shear patch should produce strain corresponding to $\gamma_{\text{xz}}^{*,\text{l}}=-d_{15}E_{z}$. On application of an electric field along the z-direction, the right piezoceramic patch should deform positively whereas the left patch should deform negatively. The combination of these two deformations will produce a global torsion of the bimorph transducer, since the patches are identical (have the same shear modulus), bonded to each other and the transverse shear strain should be continuous at the left/right patch interface.[11]

## 3. Results and discussion

### 3.1. Microstructural characterization

**Figure 3.**
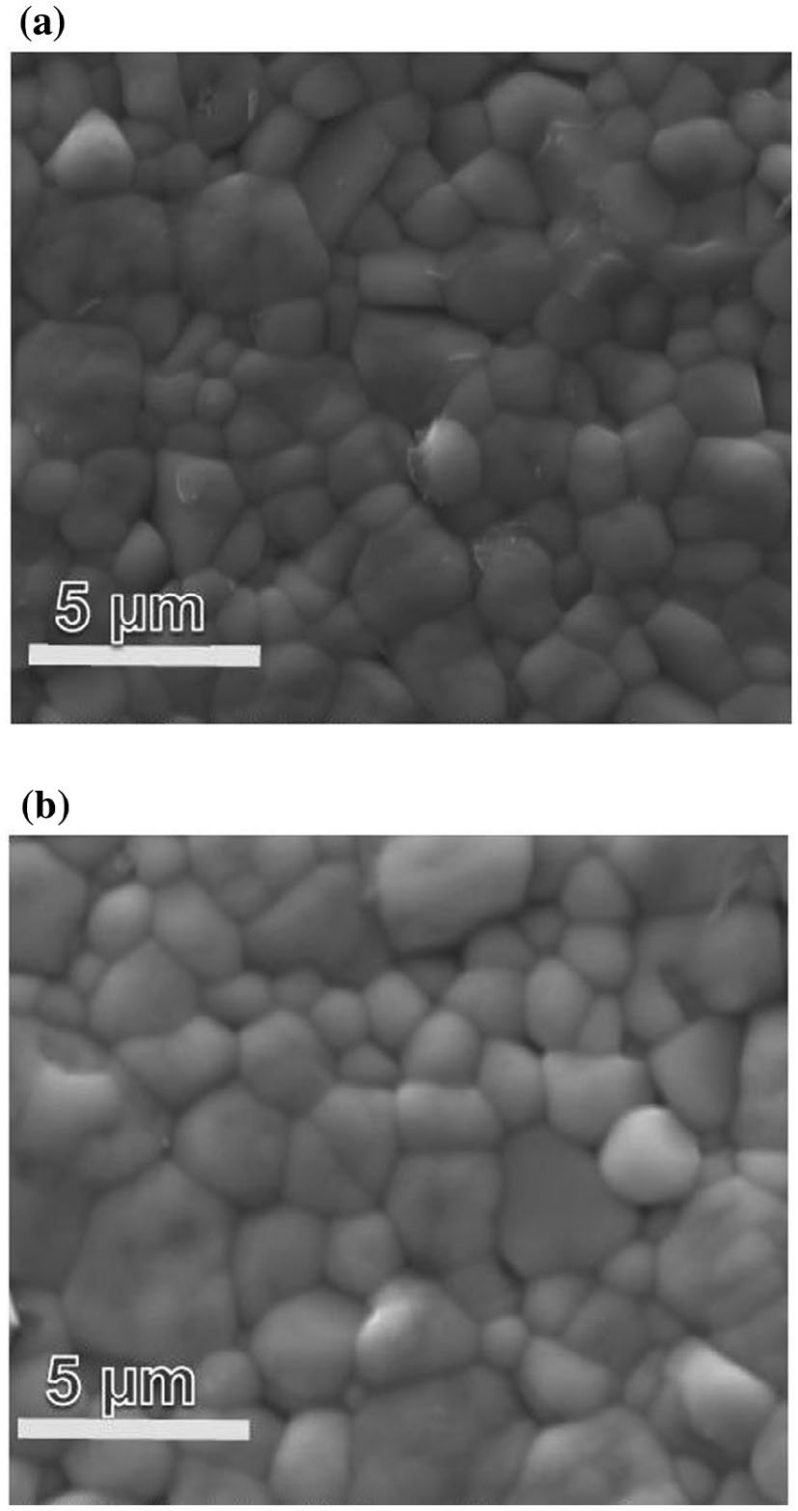
SEM images of (a) NBT-BT and (b) NBT-BT-Mn piezoceramics.

The scanning electron microscopy images (SEM, FEI Quanta 600 FEG, Hillsboro, OR, USA) of the pure NBT-BT and NBT-BT-Mn piezoceramics are compared in Figure 3(a) and 3(b), respectively. The grain size was measured using the line intercept method. The Mn-doped NBT-BT piezoceramic was found to exhibit enlarged grain size. The average grain size of NBT-BT and NBT-BT-Mn grains were approximately 1.54 μm and 1.73 μm, respectively. The densities of the ceramic samples were measured using Archimedes principle. NBT-BT-0.08 wt%Mn piezoceramic material was found to have higher relative density with 99% (5949 kg m^–3^) than NBT-BT ceramics which have a relative density of 97% (5848 kg m^–3^); the higher density is an indicator of the reduced porosity of NBT-BT-0.08 wt%Mn piezoceramics.

**Figure 4.**
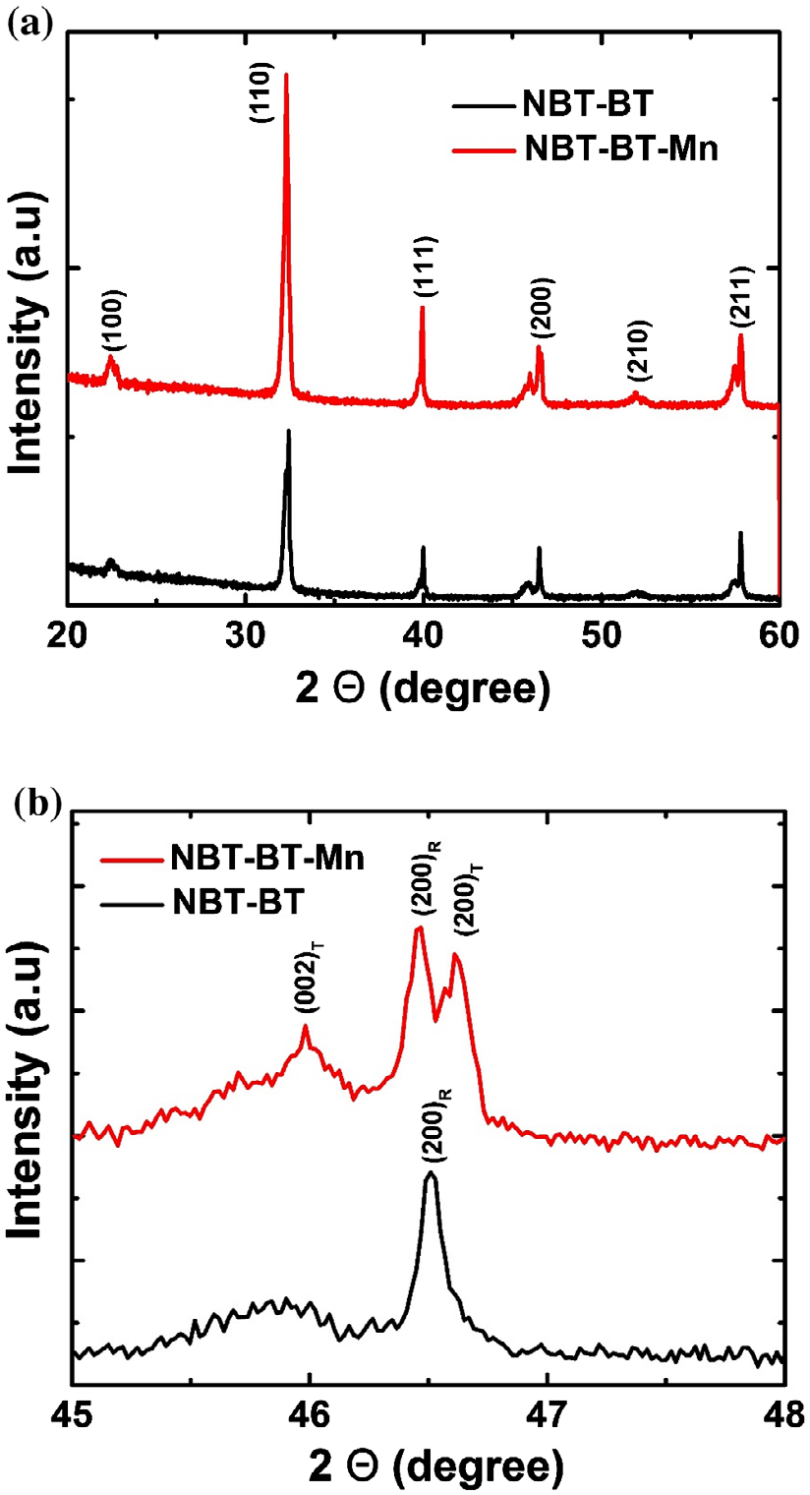
(a) XRD patterns of NBT-BT and NBT-BT-Mn piezoceramics, and (b) fine structure of the (200) peak.

Figure 4(a) and (b) compare the X-ray diffraction (XRD, D8 Advanced, Bruker) patterns of the pure NBT-BT and NBT-BT-Mn piezoelectric ceramics. Along the {200} Bragg reflection of pure NBT-BT ceramic sample, an intense diffraction peak was encountered near 2*θ* = 46.510°. However, along the pseudocubic {200} Bragg reflection of Mn-modified NBT-BT ceramic sample, the diffraction peaks were found near 2*θ* = 45.980°, 2*θ* = 46.470° and 2*θ* = 45.610°; these XRD reflections (002)_T_, (200)_R_ and (200)_T_ revealed the coexistence of rhombohedral and tetragonal phases in the Mn-NBT-BT ceramics.

**Figure 5.**
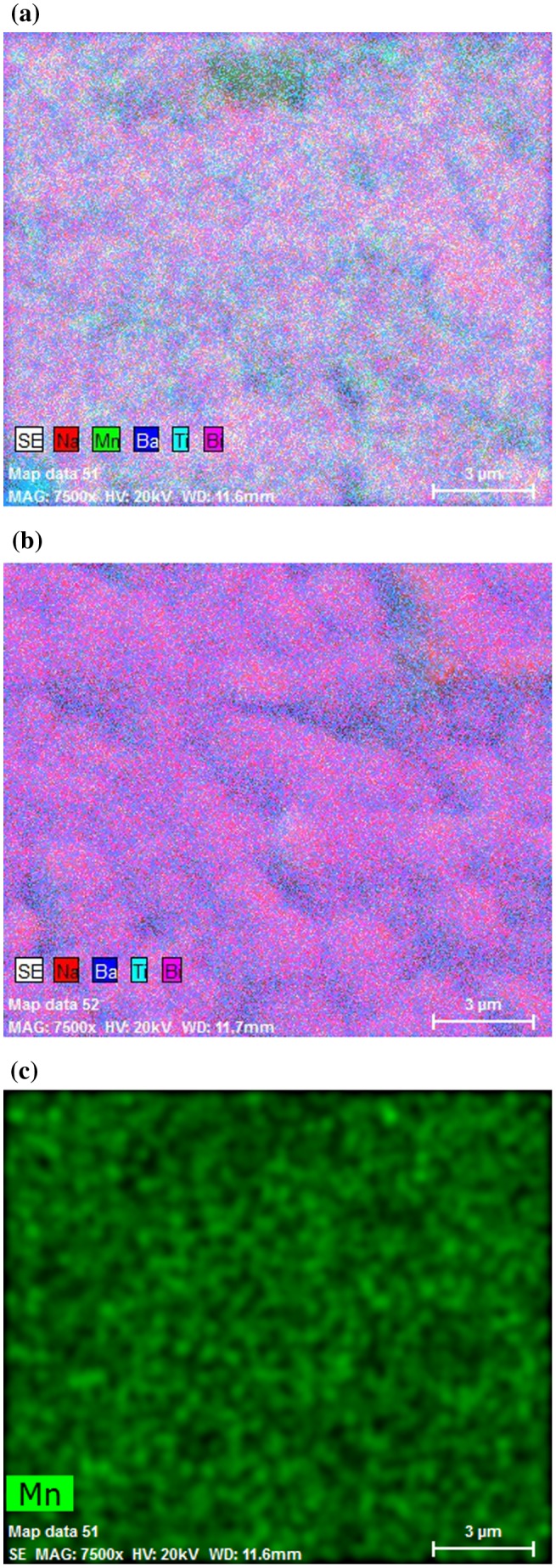
EDS elemental map comparison of the NBT-BT-Mn (a) and NBT-BT (b), EDS map of Mn in Mn-modified NBT-BT ceramic.

Energy dispersive X-ray spectroscopy (EDS) was used to further confirm the composition distribution in NBT-BT-Mn and NBT-BT ceramic samples. Figure 5 presents the EDS elemental mapping comparison of the NBT-BT-Mn and pure NBT-BT samples, and the EDS image of the distribution Mn element in Mn modified NBT-BT ceramic. EDS images confirmed that Mn element was present within the NBT-BT grains. The SEM images of the measured areas where the mappings were conducted are presented in Figure 3(a) and (b).

**Figure 6.**
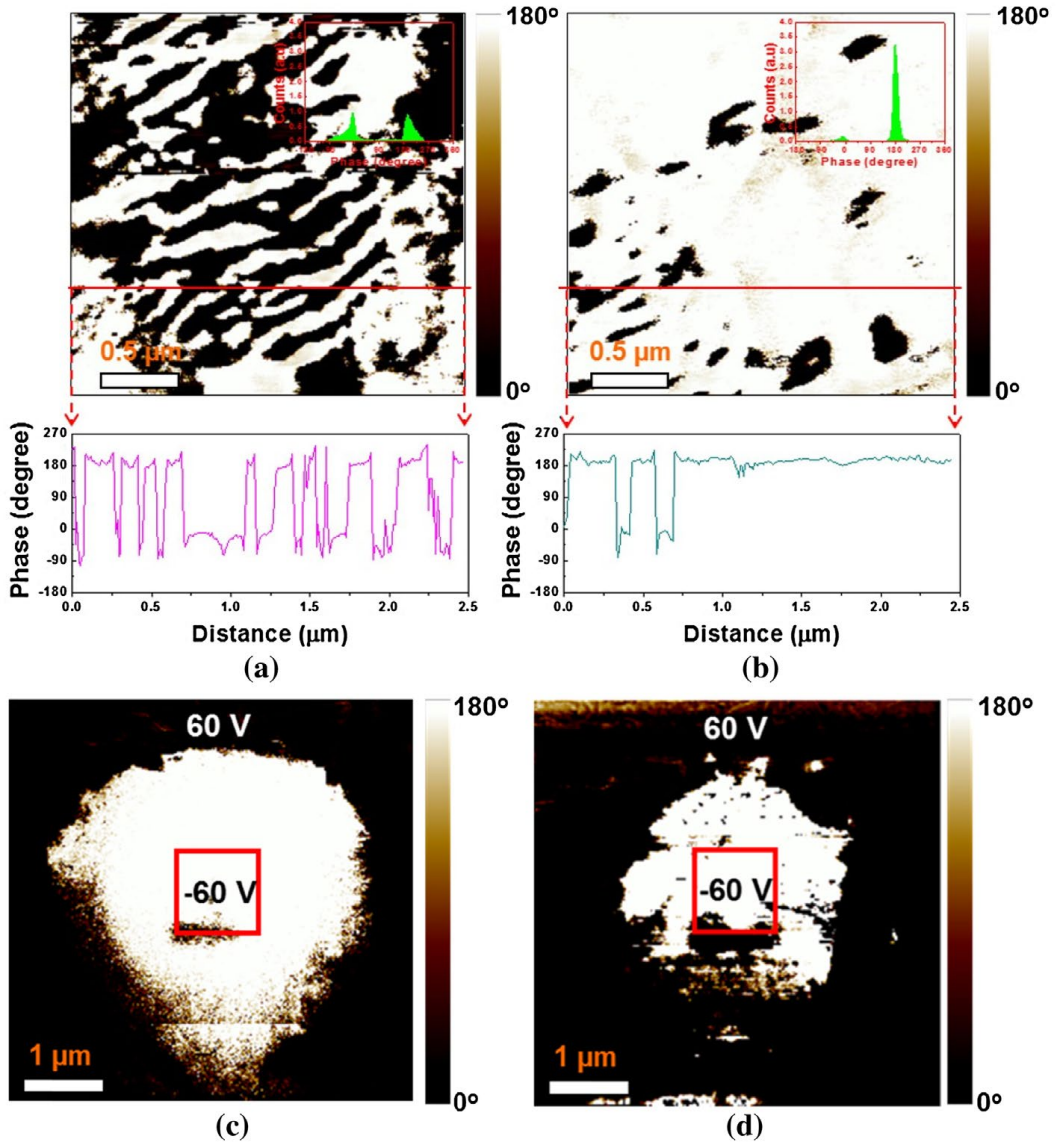
PFM image comparisons of NBT-BT and NBT-BT-Mn piezoceramics: (a) phase image of NBT-BT-Mn, (b) phase image of NBT-BT, (c) phase image of NBT-BT-Mn after polarization switching, and (d) phase image of NBT-BT after polarization switching.

Piezoresponse force microscopy (Bruker Dimension Icon, Billerica, MA, USA) phase images of the NBT-BT and NBT-BT-Mn specimens are illustrated in Figure 6(a) and (b). It can be seen from these figures that NBT-BT-Mn exhibited reduced domain size as compared to the NBT-BT specimen. The smaller domain size enhances the piezoelectric response of a system.[17] To evaluate the domain switching behavior, DC bias was applied on the surface of each specimen with –60 V and 60 V in inner and outer region of the red square (1 μm^2^ size) as drawn in Figure 6(c) and 6(d). It was observed that the NBT-BT specimen included partially un-switchable region in the switched area, which could be attributed to the domain wall pinning due to the presence of the oxygen vacancies and other point defects. Oxygen vacancies diffuse into the high stress regions of domain walls and clamp the domain wall motion.[18] The NBT-BT-Mn specimen showed a clear polarization switching behavior. The ease of polarization switching in NBT-BT-Mn can be attributed to the smaller domain size which in turn facilitates the poling resulting in enhanced piezoelectric coefficient.

**Figure 7.**
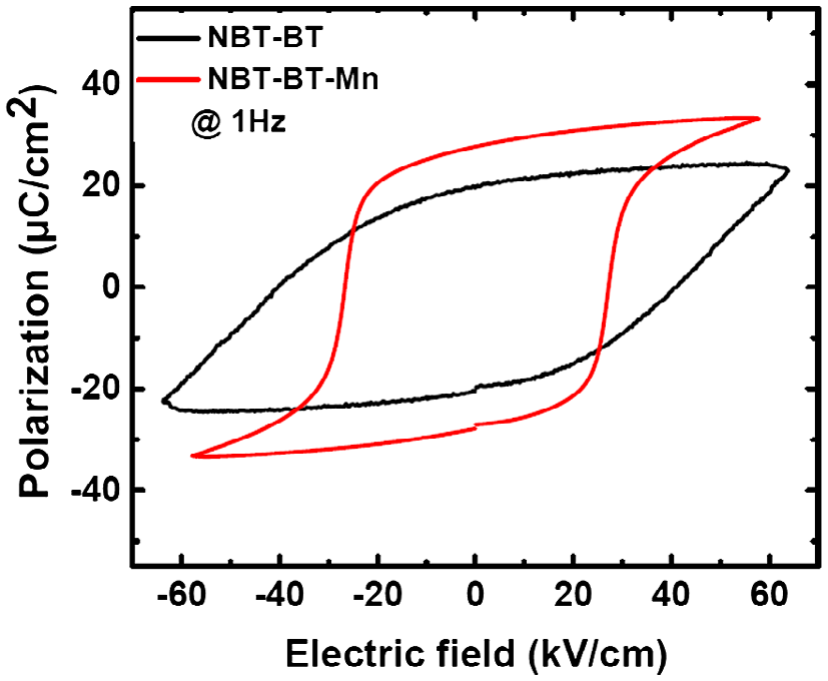
Polarization hysteresis loops for Mn modified (red curve) and pure NBT-BT (black curve) piezoceramics under E ≤ 50 kV cm^–1^.

Additionally, polarization hysteresis (P-E) measurements on the Mn-modified and pure NBT-BT ceramic samples were conducted at 1 Hz (Figure 7). Mn modified NBT-BT sample showed near complete polarization switching under E ≤ 50 kV cm^–1^, whereas the P-E loop of the pure NBT-BT sample was not completely saturated under E ≤ 50 kV/cm. The results of these P-E loops are consistent with the results obtained by the polarization switching using the PFM.

### 3.2. Piezoelectric shear-mode characterization of lead-free NBT-BT-Mn ceramics

In order to investigate the shear-mode piezoelectric properties, the samples were cut into rectangular bars. The silver electrodes were applied on the flat faces normal to the length of the bars. These bars were poled with an applied voltage ranging from 1.45 kV mm^–1^ to 1.63 kV mm^–1^ at room temperature. Next, these electrodes were removed followed by new silver electrodes on the large faces for measuring the *d*_15_ thickness shear-mode properties.

The experimental piezoelectric shear strain *d*_15_ and electromechanical coupling *k*_15_ coefficients of the NBT-BT and NBT-BT-Mn ceramic samples were determined from resonance and anti-resonance frequencies measured using an impedance analyzer (HP4194A, Hewlett Packard, USA), following the Institute of Electrical and Electronic Engineers (IEEE) standards.[19–22]

The shear-poled pure NBT-BT piezoceramic sample had a dimension of 8.58 × 2.38 × 0.36 mm^3^, whereas the NBT-BT-Mn samples had dimension of 13.77 × 3.60 × 0.54 mm^3^. The measured resonance *f*_r_ and anti-resonance frequencies *f*_*a*_ of the NBT-BT piezoceramic shear patch were 3.5 and 3.9 MHz, respectively. On the other hand, *f*_*r*_ and *f*_*a*_ frequencies of the NBT-BT-Mn piezoceramic shear patch were 2.6 and 3.1 MHz, respectively.[23]

Table 1 compares the piezoelectric *d*_15_ shear properties of the lead-free NBT-BT-Mn, pure NBT-BT, and lead-based PIC 255 piezoceramics. It can be seen that 0.08 wt% MnO_2_ substitution improved the piezoelectric shear strain coefficient *d*_15_ from 230 pC/N to 305 pC/N. Mn addition up to 0.1 wt% improves piezoelectric strain coefficient *d*_33_, and electromechanical coupling coefficient and dielectric permittivity of BNT-BT ceramics.[16] PFM analysis revealed that NBT-BT-Mn had reduced domain size as compared to the NBT-BT specimen and the smaller domain size strengthens the piezoelectric response of a system.[17] The electromechanical shear coupling *k*_15_ was found to be 0.65 as compared to the 0.51 for pure NBT-BT. The transverse shear elastic constant, *c*_55_, and the piezoelectric shear stress coefficient, *e*_15_, was found to be 38.34 GN m^–2^ and 11.6 C m^–2^ for NBT-BT-Mn respectively. For NBT-BT, the parameters *c*_55_ and *e*_15_ were found to be 34 GN m^–2^ and 7.82 C m^–2^ respectively. It should be noted that the lead-free NBT-BT-Mn piezoceramics synthesized in this study was found to exhibit almost the same piezoelectric shear stress *e*_15_ coefficient as the lead-based PIC 255 (Physik Instrumente, Karlsruhe, Germany) commercial piezoceramics.

**Table 1.** Comparison of piezoelectric *d*_15_ shear related properties of NBT-BT-Mn ceramics to the pure NBT-BT and lead-based PIC 255 piezoceramics.

Material	*d*_15_ (pC/N)	*k*_15_ (%)	*d*_33_ (pC/N)	*k*_33_ (%)	*k*_31_ (%)	*c*_55_ (GN m^–2^)	*e*_15_ (C m^–2^)
NBT-BT	230	51	160	49.8	20	34.00	7.82
PIC 255	550	66	400	69	35	22.26	11.90
NBT-BT-Mn	305	65	195	59	28	38.34	11.60

The shear mode (*d*_15_ effect) can be expressed through the piezoelectric constitutive equations as:

(2.1) $$\left\{\begin{array}{c} \sigma_{xz}\\ D_{z} \end{array}\right\}=\left[\begin{array}{cc} c_{55} & -e_{15}\\ e_{15} & \varepsilon_{11}^{T} \end{array}\right]\left\{\begin{array}{c} \gamma_{xz}\\ E_{z} \end{array}\right\}$$

which can be simplified as:

(2.2) $$\sigma_{xz}=c_{55}\gamma_{xz}-e_{15}E_{z}$$

where *σ*_*xz*_ and *γ*_*xz*_ are the transverse shear stress and strain; *D*_*z*_ and *E*_*z*_ are the transverse electric displacement and field; $\varepsilon_{11}^{T}$, *e*_15_ and *c*_*55*_ are the dielectric constant at constant (free) shear stress, piezoelectric transverse shear stress constant and elastic transverse shear constant, respectively. The transverse shear force $Q_{z}^{*}$ produced by the piezoceramic patch (shown in Figure 1) can be expressed as:

(2.3) $$Q_{z}^{*}=b\int_{-h}^{h}e_{15}E_{z}dz$$

From Table 1, Equations (2.2) and (2.3), it can be concluded that the lead-free NBT-BT-Mn piezoceramics synthesized in this study exhibited almost the same transverse shear stress and force as the lead-based commercial PIC 255 piezoceramics. Lead-free NBT-BT-Mn piezoceramic material had *k*_15_ ~ 0.65 which is similar to that of the lead-based PIC 255 piezoceramic material with *k*_15_ ~ 0.66.

### 3.3. Evaluation of the rate of twist

To evaluate the rate of twist produced by the lead-free piezoceramic *d*_15_ torsion transducer, the transverse deflections under different applied AC voltages at 10 Hz were measured using laser scanning vibrometer. The displacement values were averaged by the laser scanning vibrometer software over the measured surface of the experimental benchmark. Piezoelectric torsion actuation measurements conducted over quasi-static frequencies up to 20 Hz can be regarded as static.[13] In addition, quasi-static torsion actuation has the advantage of exhibiting negligible hysteresis behavior.[13] The rate of twist α_max_ was post-processed from the measured transverse deflections using Equation (3):

(3) $$\alpha^{\max}=\frac{u_{z}^{\max}}{Lb}$$

where $u_{z}^{\text{max}}$, *L* and *b* are the maximum tip transverse deflection, the length and half width of the torsion actuator, respectively.

**Figure 8.**
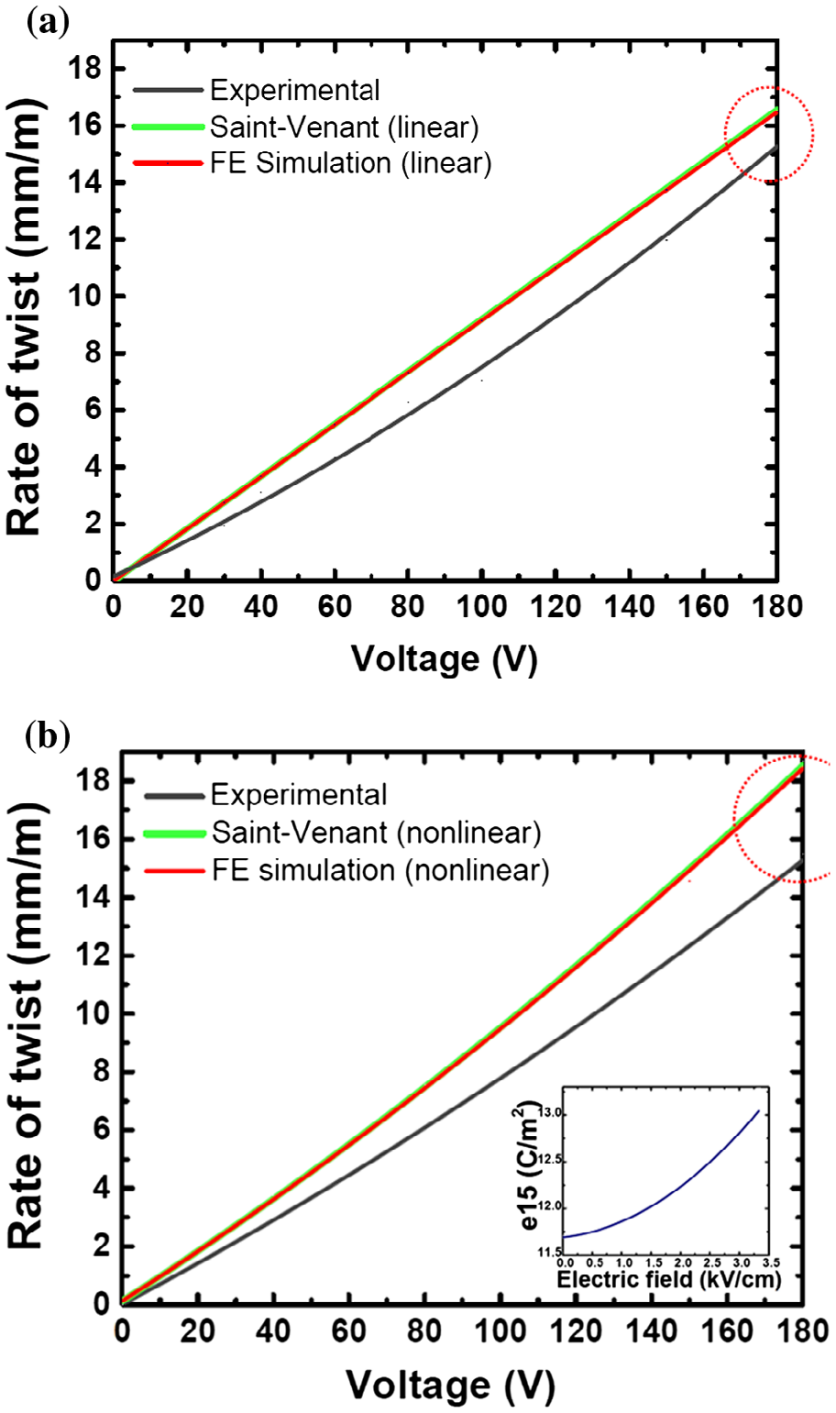
Comparison of the experimental rate of twist, and mathematical and finite element simulations when the nonlinearity of shear-mode piezoceramics is ignored (a), or included (b) in the computations.

Mathematical formulations of the present bimorph *d*_15_ torsion transducer are well established.[14,15] The mathematical analysis is based on the Saint-Venant torsion theory which is a formulation of the electromechanically coupled problem in terms of a stress function and of the electric potential and represents an exact solution of the specific three-dimensional problem. In this study, these mathematical formulations are effectively used to model the rate of twist produced from the samples.[15] Here, we provide the results of the mathematical solutions to show the validity of the experiments. In order to verify the experimental results, the experimental values of the rate of twist were compared to that obtained from the Saint-Venant type solutions and three-dimensional finite element computations by using the commercial code ABAQUS^®^ (Figure 8).

For the FE computations, C3D20E piezoelectric quadratic (20 nodes) brick elements were used with 110 elements along the length, 72 elements along the width, and four elements through the thickness, leading to a total number of 3960 elements and 185,265 nodes. The opposite polarization of two piezoceramic bars forming the actuator is implemented by changing the sign of the piezoelectric stress coefficients as negative. For this purpose, two different material properties and sections were assigned to the two rows of the lead-free piezoceramic torsion actuator. The electromechanical material properties applied in the finite element computations for the piezoceramic material NBT-BT-Mn are provided in supplementary information. All nodes at the top surface of the torsion actuator have zero potential and the electric potential at all nodes at the bottom horizontal surface is set to the applied potential V.

In order to compute the nonlinear responses, the finite element computations and the analytical solutions were updated by taking the piezoelectric nonlinearity into account.[23] The piezoelectric nonlinearity of *d*_15_ shear-mode piezoceramics is related to the domain wall motion. The non-180° domain wall motion enhances the piezoelectric shear strain coefficient and is the source of nonlinearity.[24–26] The rate of twist shown in Figure 8 exhibits linear behavior until some threshold electric field which corresponds to the potential energy barrier of the non-180° domain walls of ferroelectric ceramics. When the applied electric field is higher than the threshold electric field, the non-180° domain walls start to move dynamically and the rate of twist demonstrates nonlinearity.[25,26] It is known that *d*_15_ shear-mode piezoceramics experience considerably larger piezoelectric nonlinearity than that in the *d*_31_ mode and the effect of increase of vibration amplitude on elastic nonlinearity is much smaller.[25,27] Elastic shear *c*_55_ constant nonlinearity of *d*_15_ shear-mode piezoceramics arises from the high stress developed in the material if *d*_15_ shear-mode piezoceramics driven by voltages near resonance frequencies,[28,29] and they have small dependence on the strain.[27] This type of elastic shear constant nonlinearity may influence the design of ultrasonic devices such as ultrasonic motors, which utilize *d*_15_ shear-mode soft piezoceramics near the resonance frequency excitation; however, piezoelectric *d*_15_ shear constant nonlinearity is the main source of nonlinearity experienced by the hard type piezoceramic *d*_15_ shear-based actuators under the quasi-static excitation frequency.[27,29–32] NBT-BT-Mn and NBT-BT piezoceramics are hard type piezoceramics.

The rate of twist for the finite element solution is computed from the vertical displacement *U*_*z*_ values. For this purpose, two cross-sections located in the vicinity of the axial center of the torsion actuator are taken into consideration; namely at $X=\left(L\pm \Delta X\right)/\;2$, and the rate of twist is computed as [15]:

(4) $$\alpha^{FE}=\frac{U_{z}\left(\text{X}=\frac{\text{L}+\Delta \text{X}}{\text{2}},\;\text{Y}=-b,\;\text{Z}=0\right)-U_{z}\left(\text{X}=\frac{\text{L}-\Delta \text{X}}{\text{2}},\;\text{Y}=-b,\;\text{Z}=0\right)}{b\Delta \text{X}}$$

The rate of twist values presented in Table 2 show the correlation of the experimental rate of twist with the analytical solution and the finite element computations at an applied voltage of 40, 80 and 150 V. The Saint-Venant type solutions are in very good agreement with the three-dimensional finite element computations and their curves overlap (Figure 8). The experimental rate of twist values at 40 and 150 V were 3.110 (mm m^–1^) and 11.985 (mm m^–1^), respectively. The deviation of the experimental results from the nonlinear theoretical computations is approximately 17%. Since the transverse deflection values obtained from the laser scanning vibrometer were averaged over the measured surface area of the actuator, the experimental transverse deflection and rate of twist values were lower than the theoretical calculations. The stiffness of the clamp of the experimental benchmark may have influenced the experiments and caused these deviations between the experimental and theoretical results. The analytical and finite element models are independent from the cantilever boundary condition applied in the experiment. Nevertheless, these deviations are quite reasonable and good enough to prove the effectiveness of the lead-free piezoceramic torsion actuator for application. The first three eigenfrequencies of the torsion transducer were also determined by the finite element simulations as 3,211 Hz (first transverse x–z bending mode), 10,273 Hz (first torsion mode) and 19,680 Hz (second transverse x–z bending mode), respectively.

**Table 2.** Rate of twist (mm m^–1^) comparisons between the experimental results, analytical and finite element computations (ABAQUS^®^).

Applied voltage	Experimental (nonlinear)	Analytical (linear)	ABAQUS^®^ (linear)	Analytical (nonlinear)	ABAQUS^®^ (nonlinear)
40 V	3.110	3.680	3.638	3.777	3.733
80 V	6.262	7.362	7.276	7.602	7.514
150 V	11.985	13.803	13.643	14.572	14.402

The effective rate of twist for piezoelectric torsion actuation was calculated by dividing the rate of twist angle by the applied voltage. Table 3 summarizes the performance (in terms of effective rate of twist values) of the lead-free NBT-BT-Mn piezoceramic torsion actuator and the lead-based (PIC 255) piezoceramic torsion actuators.[11,13] The effective rate of twist value of the lead-free benchmark was 0.08 mm m^–1^ V^–1^ under quasi-static 150 V drive. On the other hand, the maximum effective rate of twist values reported for lead-based systems as reported in [11] and [13] were 0.014 and 0.034 mm m^–1^ V^–1^, respectively. Thus, the lead-free piezoceramic torsion actuator developed in this study exhibited relatively higher effective rate of twist values.

**Table 3.** Performance comparison between NBT-BT-Mn piezoceramic torsion transducer and lead-based benchmarks reported in [11] and [13] in terms of effective rate of twist.

Evaluation parameter	Present work	[11]	[13]
Effective rate of twist (mm m^–1^ V^–1^)	0.08 (at 150 V)	0.0142	0.0344

## 4. Conclusions

A lead-free NBT-BT-Mn piezoceramic *d*_15_ shear-induced torsion actuator with enhanced stress coupling was developed and evaluated in terms of generated rate of twist under quasi-static operation condition. In comparison to lead-based shear piezoceramics, the lead-free NBT-BT-Mn shear-mode piezoceramic material was found to exhibit high piezoelectric shear stress coefficient, electromechanical shear coupling coefficient and piezoelectric transverse shear actuation force. As the result of PFM analysis, NBT-BT-Mn ceramic material was found to exhibit reduced domain size which has an enhancing effect on piezoelectric response. Improved elastic shear *c*_55_ coefficient with enhanced piezoelectric shear strain *d*_15_ constant resulted in increased piezoelectric shear stress *e*_15_ coupling coefficient. The lead-free piezoceramic torsion transducer produced higher effective rate of twist (0.08 mm m^–1^ V^–1^). The experimental results were verified by Saint-Venant type mathematical solutions and three-dimensional finite element computations. They showed reasonable agreement. This lead-free piezoceramic torsion actuator can be effectively used in preventing and controlling torsional deformation in helicopter blades, robot arms and flexible space structures by taking into account the environmental protection requirements.

## Disclosure statement

No potential conflict of interest was reported by the authors.

## Supplemental data

Supplemental data for this article can be accessed here http://dx.doi10.1080/14686996.2016.1254569.

## Funding

This work was supported by the Austrian Science Fund (FWF) in the framework of Erwin Schrödinger Fellowship under grant [J-3620-N30]; by the Air Force Office of Scientific Research (AFOSR) under grant [FA9550-14-1-0376]; by the Office of Naval Research under grant [N00014-14-1-0158] and by the Office of Basic Energy Science, Department of Energy under grant [DE-FG02-06ER46290].

## Supplementary Material

suppl_data.zip
