# Supplementary material for: Enhanced torsional actuation and stress coupling in Mn-modified 0.93(Na0.5Bi0.5TiO3)-0.07BaTiO3 lead-free piezoceramic system
Source: Sci Technol Adv Mater. 2017 Jan 9;18(1):51–9. doi: 10.1080/14686996.2016.1254569 (PMC5256243; doi:10.1080/14686996.2016.1254569)
Supplement: suppl_data.zip [file tsta_a_1254569_sm3593.zip › suppl_data/Supplementary Material_STAM.docx]

**Enhanced torsional actuation and stress coupling in Mn-modified 0.93(Na_0.5_Bi_0.5_TiO_3_)-0.07BaTiO_3_ lead-free piezoceramic system**

Pelin Berik^a^, Deepam Maurya^a^, Prashant Kumar^a^, Min Gyu Kang^a^, Shashank Priya^a^

*^a^Center for Energy Harvesting Materials and Systems (CEHMS),*

*Bio-inspired Materials and Devices Laboratory (BMDL), Virginia Tech, 24061, USA*

^a^ pelin.berik@gmail.com, ^a^ [mauryad@vt.edu](mailto:mauryad@vt.edu), ^a^ [pkumar14@vt.edu](mailto:pkumar14@vt.edu), ^a^ [mgkang@vt.edu](mailto:mgkang@vt.edu),

^a^ spriya@vt.edu

*(Received XX Month Year; final version received XX Month Year)*

**Supplementary Information**

Figure S1a, Figure S1b and Figure S1c show the frequency dependence of the impedance and phase spectra of *d*_15_ shear poled 0.93NBT-0.07BT ceramic with composition near MPB (morphotropic phase boundary) and Mn modified 0.93NBT-0.07BT ceramic, respectively. It can be seen that the phase angle is negative and not exactly ±90^0^ as expected for fully poled sample. Similar behavior of the impedance phase angle curve is encountered in [S1] for the thickness *d*_33_ poled 0.93NBT-0.07BT ceramic material. A phase angle determines how much the current leads or lags the voltage in a circuit. Current leads voltage by -90^0^ in a purely capacitive circuit, whereas mixed resistive and capacitive impedance will have a phase angle somewhere between 0^0^ and -90^0^ [S2, S3, S4].

**
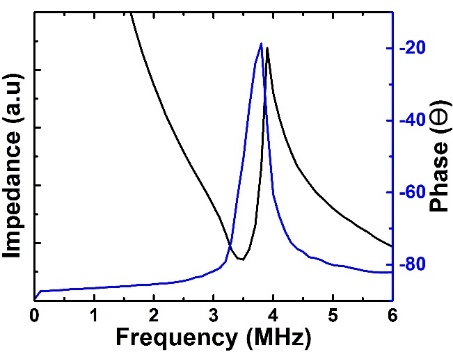
**
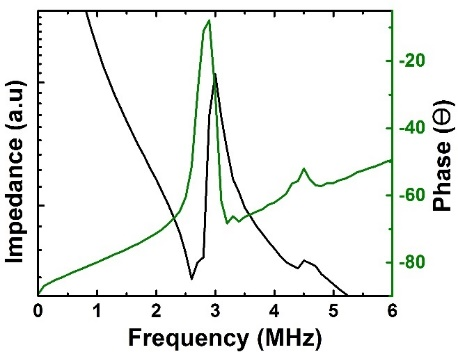




**(c)**

**(a)**

**(b)**

Figure S1: (a) Impedance/Phase spectra of *d*_15_ shear poled 0.93NBT-BT-0.07BT ceramic, (b) Impedance/Phase spectra of *d*_15_ shear poled Mn-modifed 0.93NBT-BT-0.07BT ceramic, and (c) Comparison of phase spectrum versus frequency of pure and Mn-modified 0.93NBT-BT-0.07BT.

The electromechanical parameters of NBT-BT-Mn poled along the x-axis that were used in the simulations are presented in Appendix S1. The mechanical properties of NBT-BT-Mn ceramics indicate that they are hard type of piezoceramics. NBT-BT-Mn piezoceramics have very high Young’s moduli and shear moduli like other type of BNBT based ceramics [S5, S6]. For this reason, NBT-BT-Mn ceramic material is able to produce transverse shear actuation force comparable to that of lead-based shear-mode piezoceramics.

**References**

[S1] Parija B, Badapanda T, Sahoo PK, et al. Structural and electromechanical study of Bi_0.5_Na_0.5_TiO_3_-BaTiO_3_ solid-solutions. Process and Appl Ceram. 2013;7(2):73–80.

[S2] Dubey AK, Kakimoto K, Obatab A, et al. Enhanced polarization of hydroxyapatite using the design concept of functionally graded materials with sodium potassium niobate. RSC Adv. 2014;4:24601–24611.

[S3] Gittings JP, Bowen CR, Dent ACE, et al. Electrical characterization of hydroxyapatite-based bioceramics. Acta Biomaterialia. 2009;5:743–754.

[S4] Kuphaldt TR, Lessons in Electric Circuits: Volume II – AC. Design Science License; 2003.

[S5] Takenaka T, Maruyama K, Sakata K. Bi_(1/2)_Na_(1/2)_TiO_3_-BaTiO_3_ system for lead-free piezoelectric ceramics. Jpn J Appl Phys.1991;1(30):2236-2239.

[S6] Bansal NP, Singh JP, Ko S, et al. Editors. Processing and Properties of Advanced Ceramics and Composites V: Ceramic Transactions. Wiley-American Ceramic Society. 240:272; 2013.

**Appendix S1**

Electromechanical parameters of NBT-BT-Mn poled along the x-axis that were used in the simulations

Constant Notation Value

Piezoelectric strain coefficient (pC/N) d_15_ 305

d_33_ 195

d_31_ -89

Relative dielectric permittivity $\text{ε}_{11}$ 974

$\text{ε}_{22}=\text{ε}_{33}$ 1297

Young`s moduli (GN/m^2^) E_1_ 123.34

E_2_=E_3_ 105.18

Shear moduli (GN/m^2^) G_12_=G_13_ 38.34

G_23_ 38.67

Poisson ratios $\upsilon_{12}=\upsilon_{13}$ 0.31

$\upsilon_{23}$ 0.36

Density (kg/m^3^) ρ 5949
