# Supplementary figures and images for: Enhanced torsional actuation and stress coupling in Mn-modified 0.93(Na0.5Bi0.5TiO3)-0.07BaTiO3 lead-free piezoceramic system
Source: Sci Technol Adv Mater. 2017 Jan 9;18(1):51–9. doi: 10.1080/14686996.2016.1254569 (PMC5256243; doi:10.1080/14686996.2016.1254569)

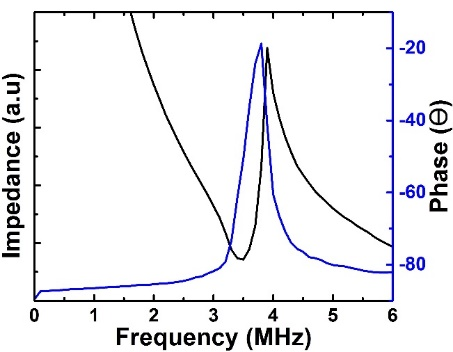

Supplement: suppl_data.zip [file tsta_a_1254569_sm3593.zip › suppl_data/FigureS1a.tif]

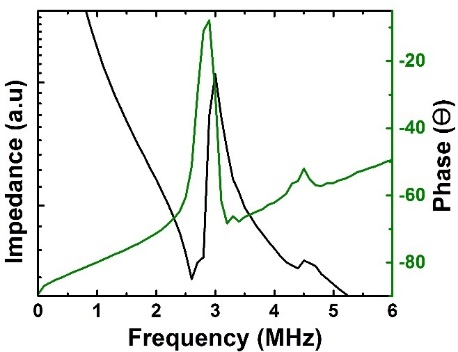

Supplement: suppl_data.zip [file tsta_a_1254569_sm3593.zip › suppl_data/FigureS1b.tif]
